# Fatty Liver Is an Independent Risk Factor for Elevated Intraocular Pressure

**DOI:** 10.3390/nu14214455

**Published:** 2022-10-23

**Authors:** Jun-Hyuk Lee, Yu-Jin Kwon, Hye Sun Lee, Jee Hye Han, Boyoung Joung, Sung Jin Kim

**Affiliations:** 1 Nowon Eulji Medical Center, Department of Family Medicine, Eulji University School of Medicine, Seoul 01830, Korea; 2 Department of Medicine, Graduate School of Hanyang University, Seoul 04763, Korea; 3 Department of Family Medicine, Yongin Severance Hospital, Yonsei University College of Medicine, Seoul 16995, Korea; 4 Biostatistics Collaboration Unit, Department of Research Affairs, Yonsei University College of Medicine, Seoul 06273, Korea; 5 Department of Internal Medicine, Division of Cardiology, Yonsei University College of Medicine, Seoul 03722, Korea; 6 Nowon Eulji Medical Center, Department of Ophthalmology, Eulji University School of Medicine, Seoul 01830, Korea

**Keywords:** intraocular pressure, fatty liver, alcoholic liver disease, insulin resistance

## Abstract

Elevated intraocular pressure (EIOP) is a major risk factor for glaucoma. Both EIOP and fatty liver share metabolic risk factors, which implies a possible link between EIOP and fatty liver. We aimed to determine the association of fatty liver with EIOP and estimate the effect of fatty liver on EIOP directly and indirectly through insulin resistance. Data from 16,240 adults who underwent health examinations at a single center were analyzed. Multiple logistic regression analyses revealed that fully adjusted odds ratio (OR) and 95% confidence interval (CI) for EIOP in the fatty liver group compared to the non-fatty liver group were 1.36 and 1.08–1.71. Alcoholic liver disease was associated with EIOP in subgroup analysis (OR = 1.80, 95% CI: 1.27–2.56). There was a linear dose–response relationship between EIOP and the severity of fatty liver. Mediation analysis revealed that the total effect of fatty liver on intraocular pressure was 0.90 (0.81–0.99), with a direct effect of 0.81 (0.71–0.90) and an indirect effect of 0.09 (0.06–0.11) through insulin resistance. Fatty liver is independently associated with EIOP. It primarily has a direct effect on intraocular pressure. This suggests that evaluation of EIOP should be considered in patients with fatty liver.

## 1. Introduction

Glaucoma can severely threaten visual function. Although not all glaucoma occurs with elevated intraocular pressure (EIOP), EIOP remains one of the major risk factors for the development of glaucoma [1]. According to a Japanese prospective cohort study, approximately 10% (7 of 75) of patients with EIOP but without visual field defects newly developed typical glaucomatous visual field defects during nine years of follow-up [2]. Therefore, early detection and management of EIOP has been emphasized to reduce the risk of glaucoma development. In addition, numerous factors, such as male sex, older age, obesity, blood pressure, heart rate, alcohol intake, smoking, hematocrit, insulin resistance, history of hypertension, and diabetes mellitus can affect EIOP [3,4,5,6]. Thus, determining and managing systemic factors related to EIOP could be a strategy for preventing glaucoma.

Fatty liver includes alcoholic fatty liver, nonalcoholic fatty liver disease (NAFLD), and fatty liver of any cause. NAFLD is the most common chronic liver disease worldwide. In 2019, the prevalence of NAFLD among the global population was estimated to be approximately 37.3% [7]. Similarly, the average prevalence of NAFLD in South Korea was estimated to be 30.3% during 2001–2014 and is rising [8]. Moreover, global economic burden from NAFLD is tremendous. In the US, between 2010 and 2014, the average medical cost per NAFLD patient was $7804 for a new diagnosis and $3789 for long-term management, which was 1.65 times more than that for patients without NAFLD [9]. Comorbidities accompanying patients with NAFLD, such as obesity, hypertension, diabetes mellitus, dyslipidemia, or cardiovascular disease [10,11,12], partly accounted for the economic burden. Insulin resistance is the most prevalent risk factor of fatty liver disease. It not only directly induces hepatic steatosis but also indirectly promotes it by increasing the secretion of proinflammatory cytokines such as interleukin (IL)-1, IL-6, and tumor necrosis factor-alpha (TNF-α) [13]. Contrarily, patients with fatty liver can also develop insulin resistance through lipotoxicity and adipose tissue inflammation, which are induced by excessive intrahepatic triglycerides in NAFLD [13,14]. Furthermore, in alcoholic fatty liver disease, insulin resistance can develop from the effects of alcohol on hepatic and nonhepatic tissue [15].

Based on evidence of the association between fatty liver and insulin resistance and the association between insulin resistance and EIOP [5,13,14], we hypothesized that fatty liver could also be related to EIOP. However, to date, there is limited evidence regarding the relationship between fatty liver and EIOP. Therefore, we investigated the relationship between fatty liver and EIOP. The direct effect of fatty liver on intraocular pressure (IOP) and its indirect effect on IOP via insulin resistance were also verified.

## 2. Materials and Methods

### 2.1. Study Population

**Figure 1:**
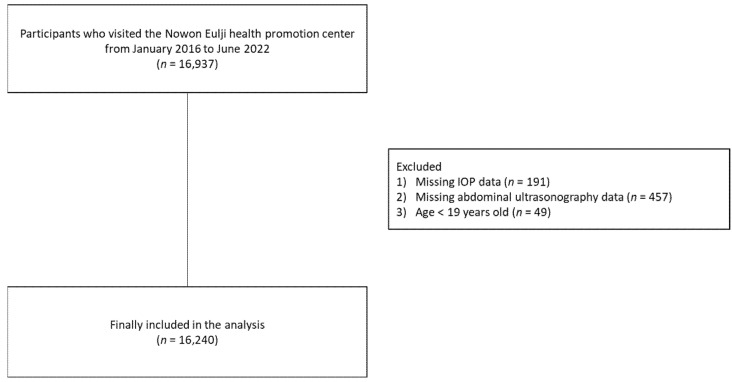
Flowchart of the study population.

This was a retrospective single-center cross-sectional study. Figure 1 shows the flowchart of the study population. From 1 January 2016 to 10 June 2022, a total of 16,937 participants visited the health promotion center of an education hospital, Nowon Eulji Medical Center, and underwent a medical health checkup after voluntarily paying for it. We finally selected 16,240 adults who completed health checkups at Nowon Eulji Medical Center after excluding (1) those without IOP data (*n* = 191), (2) those without abdominal ultrasonography data (*n* = 457), and (3) adolescents aged <19 years (*n* = 40). Participants were classified into the fatty liver group (*n* = 5786) or nonfatty liver group (*n* = 10454).

This study conformed to the ethical guidelines of the 1964 Declaration of Helsinki and its later amendments. Informed consent was waived because researchers only accessed and analyzed the deidentified data because of the retrospective study design. The Eulji University College of Medicine Ethics Committee approved the study protocol (IRB 2022-07-022).

### 2.2. Assessment of Fatty Liver

Abdominal ultrasonography imaging was performed by two experienced radiologists, using LogiQ E9 (GE Healthcare, Milwaukee, WI, USA). Images were obtained with the participants in the supine position. Fatty liver was diagnosed when there was parenchymal brightness, liver-to-kidney contrast, deep beam attenuation, and bright vessel walls on the images [16]. The severity of fatty liver was classified into mild, moderate, and severe degrees. The degrees of fatty liver was divided into 3 classes based on the degree of fat infiltration: (1) mild (a little range of fine echo in liver parenchyma with normal echo in the border of diaphragm and intrahepatic vessels), (2) moderate (moderate range of fine echo in liver parenchyma with some damage in the image of diaphragm and intrahepatic vessel), and (3) severe (vast range of fine echo in liver parenchyma hardly showing the image of diaphragm, intrahepatic vessels, and the back part of the right lobe liver) [16]. We further classified patients with fatty liver into those with (1) fatty liver with viral hepatitis infection, (2) alcoholic liver disease (ALD), and (3) NAFLD. Fatty liver with viral hepatitis infection was defined as positive results for hepatitis B surface antigen (HBsAg), or anti-hepatitis C antibody (HCV Ab), or a self-reported history of chronic hepatitis B virus (HBV) or hepatitis C virus (HCV) infection. Due to lack of information regarding the amount of alcohol intake in the self-reported questionnaire, the ALD/NAFLD index (ANI) was used to distinguish between ALD and NAFLD among the remaining patients with fatty liver. The formula for calculating ANI is as follows [17]:

ANI = −58.5 + 0.637 × (mean corpuscular volume (MCV)) + 3.91 × (aspartate aminotransferase (AST)/alanine aminotransferase (ALT)) − 0.406× (body mass index (BMI)) + 6.35 (for men).

ALD was defined as an ANI ≥ 0. NAFLD was defined as nondrinking or ANI < 0. Among 5786 people with fatty liver, the number of people with viral hepatitis, ALD, and NAFLD was 136, 1024, and 4626, respectively.

### 2.3. IOP Measurement

IOP measurements were performed by two well-trained paramedical assistants, using a Topcon CT-80 noncontact tonometer (Topcon Medical Systems, Paramus, NJ, USA), without applying topical anesthetics. Measurements were taken in the morning (08:00 a.m. to 11:00 a.m.) to minimize the diurnal fluctuation effect on IOP. The average of two successive measurement values were used to calculate IOP. EIOP was defined as IOP ≥22 mmHg.

### 2.4. Assessment of Insulin Resistance

Insulin resistance was assessed using the homeostatic assessment model for insulin resistance (HOMA-IR). HOMA-IR is the most commonly used surrogate marker to assess insulin resistance in the clinical field because the hyperinsulinemic–euglycemic clamp technique, a gold standard method to assess insulin resistance, is an invasive and time-consuming method. HOMA-IR was calculated using the following equation [18]:

HOMA-IR = (fasting plasma glucose (FPG) in mg/dL × fasting serum insulin in μIU/mL)/405

HOMA-IR ≥ 2.5 was defined as an indicator of insulin resistance [19].

### 2.5. Covariates

Participants responded to a questionnaire on their medical history, including hypertension, diabetes mellitus, dyslipidemia, stroke, and cardiovascular disease, as well as a history of infectious diseases, such as tuberculosis or HBV infection. A medical doctor at the Health Promotion Center confirmed their medical history and history of infectious diseases through person-to-person interviews.

Information about smoking status, drinking status, and physical activity of the participants was also obtained by responding to the questionnaire and by an interview with the medical doctor. Smoking status was classified as never-smoker, former smoker, or current smoker. A never-smoker was defined as a person who had never smoked or had smoked <100 cigarettes in their lifetime. A former smoker was defined as a person who had quit smoking at the time of visit and had smoked ≥100 cigarettes in their lifetime. A current smoker was defined as a person who was smoking at the time of visit and had smoked ≥100 cigarettes in their lifetime. Drinking status was divided into current drinkers and nondrinkers. Participants with ≥20 min of high-intensity physical activity ≥3 times a week or ≥30 min of moderate-intensity physical activity ≥5 times a week were classified as regular exercisers, and the rest of the participants were classified as non-regular exercisers.

Height (m) and weight (kg) were measured to the nearest 0.01 cm and 0.1 kg, respectively. Body mass index (BMI) was calculated as the waist circumference (cm) measured at the midpoint between the iliac crest and the lowest rib. Systolic blood pressure (SBP, mmHg) and diastolic blood pressure (DBP, mmHg) were measured in the sitting position after at least 30 min of rest. The mean blood pressure (MBP) was calculated.

After at least 12 h of fasting, blood samples were collected from the participants. Whole blood hematocrit was analyzed using ADVIA 2120i (Siemens Healthcare Diagnostics, Deerfield, MA, USA). FPG and serum concentrations of total cholesterol, triglycerides, high-density lipoprotein (HDL) cholesterol, low-density lipoprotein (LDL) cholesterol, AST, ALT, and high-sensitivity C-reactive protein (hsCRP) were analyzed using Chemistry XPT (Siemens Healthcare Diagnostics, Deerfield, MA, USA). HBsAg and anti-HCV Ab titers were analyzed using ADVIA Centaur XPT (Siemens Healthcare Diagnostics, Deerfield, MA, USA). The concentration of glycosylated hemoglobin (HbA1c) was analyzed using Tosoh HLD-723 G7 (HLC-723, Tosoh Corp., Tokyo, Japan).

Diabetes mellitus was defined according to the following criteria: (1) FPG ≥ 126 mg/dL, (2) HbA1c ≥ 6.5%, (3) treatment with oral antidiabetic medications, or (4) treatment with insulin therapy. Hypertension was defined based on the following criteria: (1) SBP ≥ 140 mmHg, (2) DBP ≥ 90 mmHg, or (3) treatment with antihypertensive medications. Dyslipidemia was defined according to the following criteria: (1) serum total cholesterol ≥240 mg/dL, (2) serum triglyceride ≥200 mg/dL, (3) serum HDL cholesterol <40 mg/dL, (4) serum LDL cholesterol ≥160 mg/dL, or (5) treatment with lipid-lowering medications. History of HBV was defined according to the following criteria: (1) positive HBsAg test or (2) self-reported history of chronic HBV infection. A history of HCV infection was defined as a positive anti-HCV antibody test or self-reported history of chronic HCV infection.

### 2.6. Statistical Analysis

All data are presented as mean ± standard deviation for continuous variables and number (%) for categorical variables. For continuous variables such as age, BMI, waist circumference, MBP, whole blood hematocrit, FPG, serum total cholesterol, AST, ALT, and hsCRP levels, Students’ *t*-test was used to compare differences in variables between the two groups. For categorical variables, such as diabetes mellitus, hypertension, dyslipidemia, HBV, and HCV, a chi-squared test was used to compare differences in variables between the two groups.

Multiple logistic regression analyses were used to estimate the odds ratio (OR) with 95% confidence interval (CI) for EIOP in the fatty liver group compared to the non-fatty liver group. Model 1 included age, sex, and BMI. Model 2 included the variables used in Model 1 plus smoking status, drinking status, and regular exercise status. Model 3 included the variables used in Model 2 plus hematocrit, history of diabetes mellitus, hypertension, and dyslipidemia. The crude and adjusted ORs (95% CI) for EIOP according to the etiology of fatty liver were estimated using multiple logistic regression analyses. A restricted cubic spline curve was used to determine the dose–response association of the OR for EIOP with the severity of fatty liver. A mediation analysis was performed to determine how HOMA-IR, as a mediator, affected the relationship between NAFLD and EIOP. Direct effect was defined as the amount of effect directly exerted on EIOP, irrespective of the mediator (insulin resistance). Indirect effect was defined as the effect of NALFD on EIOP through insulin resistance. The total effect was calculated by summing the direct and indirect effects.

All statistical analyses were conducted using SPSS software version 25 (SPSS Inc., Chicago, IL, USA). All statistical tests were two-sided, and statistical significance was set at *p* < 0.05.

## 3. Results

### 3.1. Clinical Characteristics of the Study Population

Table 1 shows the clinical characteristics of the study population. Among the 16,240 participants, 8866 (54.6%) were men. The mean age was 46.3 ± 12.4 years. The total number of people with EIOP was 455. The fatty liver group had a higher proportion of men, current smokers, current drinkers, and patients with diabetes mellitus, hypertension, and dyslipidemia, as well as higher mean age, BMI, waist circumference, MBP, whole blood hematocrit, FPG, HbA1c, HOMA-IR, IOP, serum total cholesterol, AST, ALT, and hsCRP levels than the non-fatty liver group.

**Figure 2:**
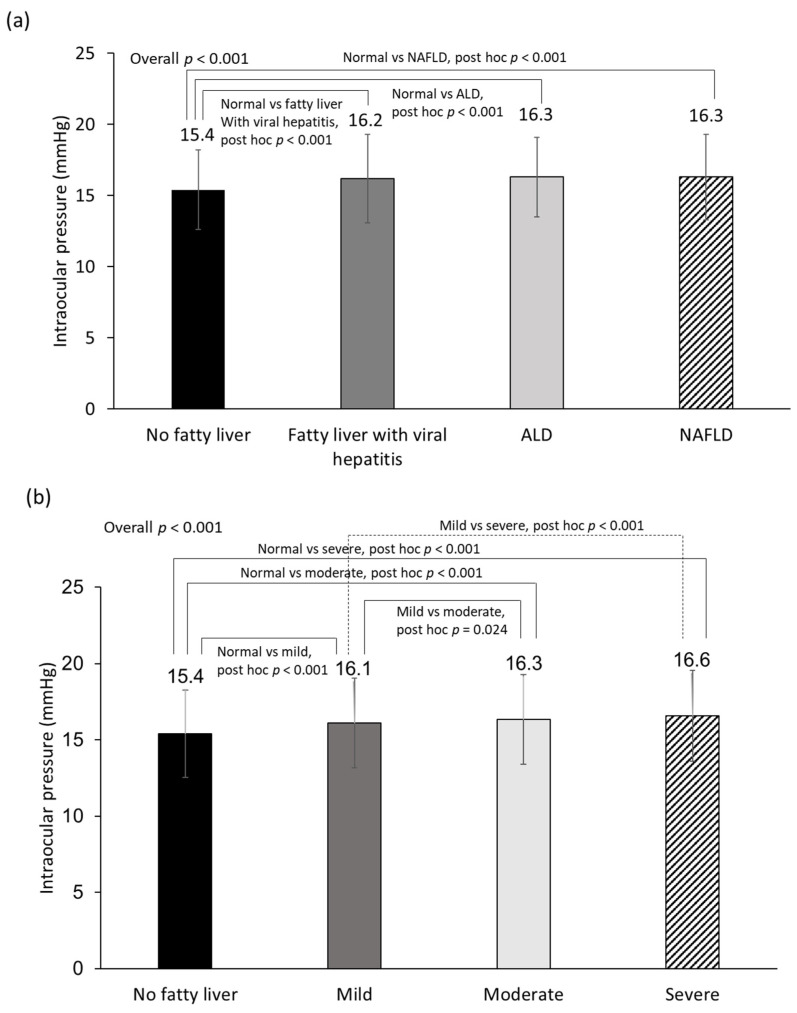
Comparison of mean intraocular pressure level according to the etiology of fatty liver (**a**) and the severity of fatty liver (**b**). Abbreviations: ALD, alcoholic liver disease; NAFLD, nonalcoholic fatty liver disease. Analysis of variance was performed to compare differences in intraocular pressure among groups. Post hoc analysis was performed with Bonferroni correction. *p* < 0.05 was considered statistically significant.

Figure 2a,b show a comparison of mean IOP according to the etiology and severity of fatty liver. The mean IOP levels in the non-fatty liver group, fatty liver with viral hepatitis, ALD, and NAFLD were 15.4, 16.2, 16.3, and 16.3 mmHg, respectively, which is the lowest in the non-fatty liver group. In post hoc analysis, the non-fatty liver group had significantly lower IOP than the fatty liver groups of any etiology. There was no significant difference in IOP according to the etiology of fatty liver. The mean IOP values of the mild, moderate, and severe fatty liver groups were 16.1, 16.3, and 16.6 mmHg, respectively. In the post hoc analysis, the non-fatty liver group had significantly lower IOP than fatty liver groups of any severity. The mild fatty liver group also had significantly lower IOP than the moderate and severe fatty liver groups.

### 3.2. Relationship between Fatty Liver and EIOP

Table 2 presents logistic regression analysis showing the relationship between fatty liver and EIOP. The risk of EIOP was increased in the fatty liver group compared to non-fatty liver group. The OR (95% CI) for EIOP in fatty liver group compared to non-fatty liver group was 2.24 (1.86–2.70). Similar trends occurred in multiple logistic regression models. The adjusted OR (95% CI) for EIOP in fatty liver group, compared to non-fatty liver group, was 1.45 (1.16–1.80) in Model 1, 1.47 (1.18–1.84) in Model 2, and 1.36 (1.08–1.71) in Model 3.

Table 3 shows the risk of EIOP according to the etiology of fatty liver. Compared to the non-fatty liver group, OR (95% CI) for EIOP in the fatty liver groups with viral hepatitis, ALD, and NAFLD were 1.90 (0.77–4.69), 2.55 (1.86–3.50), and 2.18 (1.78–2.66), respectively. The fully adjusted ORs (95% CI) for EIOP in fatty liver groups with viral hepatitis, ALD, and NAFLD were 1.01 (0.40–2.54), 1.80 (1.27–2.56), and 1.26 (0.98–1.61), respectively.

### 3.3. Dose–Response Relationship between EIOP and the Severity of Fatty Liver

**Figure 3:**
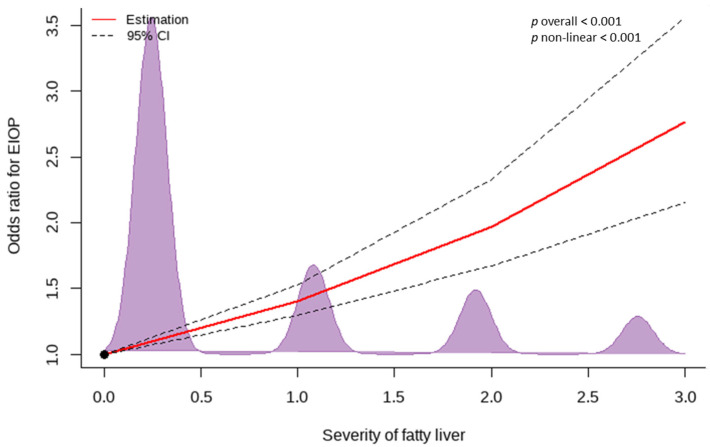
Dose–response relationship between odds ratio for EIOP and the severity of fatty liver shown as a restricted cubic spline curve. The red line indicates an estimated odds ratio. The dotted lines indicate 95% confidence intervals. The purple area indicates the density of the population. Abbreviations: EIOP, elevated intraocular pressure; CI, confidence interval. *p* < 0.05 was considered statistically significant.

Figure 3 presents a cubic spline curve showing the dose–response association of OR for EIOP with the severity of fatty liver. The density curve is shown in violet. There was a linear dose–response association between the OR for EIOP and the severity of fatty liver disease. Compared to the non-fatty liver group, the OR for EIOP increased in the mild, moderate, and severe fatty liver groups in a linear dose–response manner from mild degree to severe degree of fatty liver.

### 3.4. Insulin Resistance-Mediated Effect of Fatty Liver on IOP

**Figure 4:**
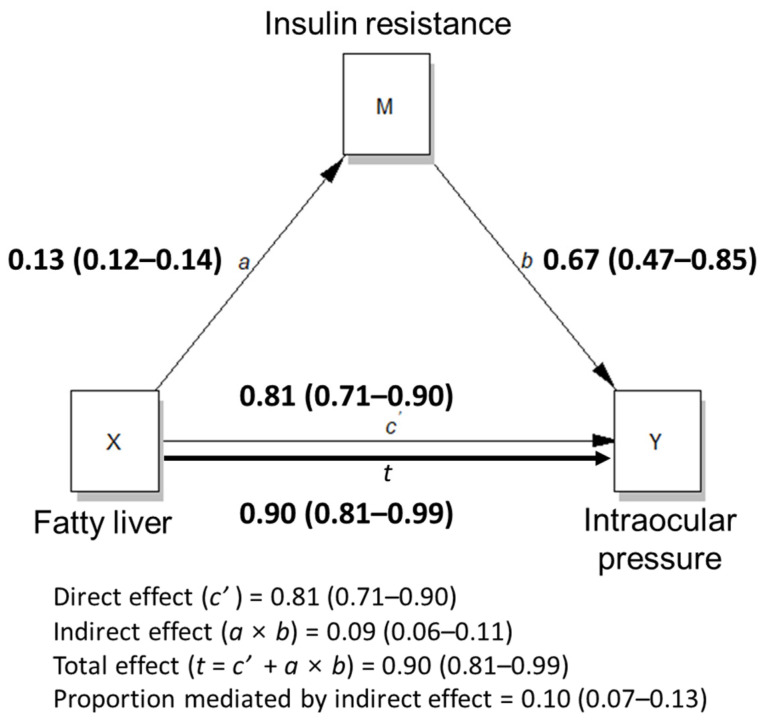
Mediation analysis on the effect of fatty liver on intraocular pressure. *a*, *b* and *c’* are path coefficients representing unstandardized regression weights and 95% confidence interval. *c’*, direct effect; *a* × *b*, indirect effect; *t*, total effect; X, dependent variable; M, mediator variable; Y, independent variable; IOP, intraocular pressure.

Figure 4 shows the total effect of fatty liver on IOP by summating the direct effect of fatty liver on IOP and its indirect effect on IOP through insulin resistance, using mediation analysis. The total effect of fatty liver on IOP was 0.90 (0.81–0.99), which means that the IOP of people with fatty liver was 0.90 mmHg higher than that of people without fatty liver, which is significant. The direct effect was 0.81 (0.71–0.90) and the indirect effect was 0.09 (0.06–0.11), which indicates that insulin resistance contributed to 0.10 (0.07–0.13) of fatty liver-related IOP.

## 4. Discussion

We found that fatty liver disease was independently associated with EIOP. The relationship between fatty liver and EIOP persisted when we adjusted for risk factors of NAFLD or IOP. In addition, there was a linear dose–response relationship between EIOP and the severity of fatty liver disease. Considering the etiology of fatty liver, ALD and NAFLD were significantly related to EIOP, although the relationship between NAFLD and EIOP was attenuated in the fully adjusted model.

Kwon et al. [20] reported that the severity of NAFLD was associated with high IOP (>15 mmHg) in South Korean adults. They also found that the more severe the fatty liver, the higher the IOP. Our study showed a significant association of EIOP with fatty liver even using the standard definition of EIOP (IOP ≥ 22 mmHg), which supports and extends the results from the previous study. Furthermore, we also determined that only ALD, but not NAFLD, showed a significant association with EIOP when we further adjusted for metabolic confounding factors, including hematocrit, history of diabetes mellitus, hypertension, and dyslipidemia. This implies that the impact of such metabolic factors on EIOP outweighs that of NAFLD.

SBP is a strong risk factor for EIOP [21,22,23]. According to a previous longitudinal prospective study, [21] for a 10 mmHg increase in SBP and DBP, there was a 0.21 mmHg and 0.43 mmHg increase in IOP, respectively. In addition, the pooled average increase in IOP associated with a 10 mm Hg increase in SBP was 0.26 mmHg, and the average increase associated with a 5 mmHg increase in DBP was 0.17 mmHg in a meta-analysis [23]. In this study, the presence of fatty liver contributed to the elevation of IOP by 0.879 mmHg. This is much higher than the effect of a 10 mmHg increase in blood pressure on IOP. Approximately 90% of the effect was directly because of fatty liver, while the indirect effect of fatty liver on IOP via insulin resistance was only 10%. This indicates that fatty liver could be one of the major risk factors for EIOP. Considering the bidirectional relationship between fatty liver and insulin resistance, the fatty liver-mediated effect of insulin resistance on IOP was also determined (Supplementary Figure S1). The percentage of indirect effect on the total effect was 0.35 (0.28–0.44). Many previous studies have verified that insulin resistance is an independent risk factor for EIOP [24,25,26]. According to the results of this study, approximately 35% of previous results may be explained by the presence of fatty liver. Therefore, improving fatty liver and reducing insulin resistance could be a strategy to manage IOP and prevent glaucoma.

Both the fatty liver groups with ALD and NAFLD were at an increased risk of EIOP. However, the significant association with EIOP remained only in ALD after adjusting for confounding factors, which suggests that more attention should be given to people with ALD, especially those with various comorbidities, including hypertension, diabetes mellitus, and dyslipidemia, for the early detection of EIOP to prevent glaucoma progression. Interestingly, there was no difference in mean IOP levels between ALD and NAFLD. In addition, although the mean IOP of alcohol drinkers was lower than that of nondrinkers (15.6 mmHg in alcohol drinkers vs. 15.8 mmHg in nondrinkers, *p* < 0.001), the OR (95% CI) for EIOP of ALD compared to NAFLD was 1.17 (0.85–1.61, *p* = 0.327) as well as the OR (95% CI) for EIOP in alcohol drinkers compared to nondrinkers was 0.86 (0.72–1.02, *p* = 0.116) (Supplementary Figure S2). There has been conflicting evidence on the effect of alcohol intake on IOP [27,28,29]. Seddon et al. [27] found that mild to moderate alcohol intake could reduce IOP. Contrarily, Song et al. [29] reported that men without open-angle glaucoma who consumed alcohol > 2 times per week and women with open-angle glaucoma who consumed alcohol > 4 times per week had increased IOP. Based on previous studies, this study suggests that the risk of EIOP is lower in drinkers than in nondrinkers in the general population, but it is increased in excessive drinkers with ALD. Follow-up studies are needed to identify the risk of EIOP based on the amount of alcohol consumed. The fatty liver group with viral hepatitis was not at risk of EIOP compared to the non-fatty liver group. The small sample of the fatty liver group with viral hepatitis may have affected the results.

There are several possible explanations for the association between fatty liver disease and EIOP. First, when there is chronic alcohol consumption or high saturated fatty acid, cholesterol, or fructose ingestion, increased gut permeability through decreased expression of intestinal regenerating family member 3 gamma (*REG3G*) as well as reduction in the levels of tight junction proteins leads to leakage of gut microbiota products/metabolites to blood vessels, which induces ectopic immune stimulation, especially in the liver [30]. Increased fatty liver-induced proinflammatory cytokines such as IL-1β, IL-18, and TNF-α can affect the development of atherosclerosis [31]. Atherosclerosis contributes to the loss of autoregulatory capacity of vessels, resulting in dysfunction of IOP and blood flow autoregulation [32]. Second, increased blood viscosity affects the elevation of both insulin resistance and IOP. Increased blood viscosity increases insulin resistance through insufficient oxidative delivery in peripheral tissue, which contributes to fatty liver [33]. The increase in blood viscosity can also elevate resistance to outflow in episcleral veins and induce a compensatory increase in blood flow to the ophthalmic artery by vasodilatation and blood pressure elevation [34]. Third, obesity can contribute to both fatty liver and IOP. The free fatty acids released from adipose tissue are absorbed into the liver and used as a source of triglyceride synthesis in the liver, resulting in hepatic steatosis [35]. Episcleral venous pressure can increase as intraorbital adipose tissue increases, resulting in reduced outflow of aqueous humor [34].

This study had several limitations. First, the IOP was measured only in the morning. Therefore, diurnal fluctuations in IOP could not be considered. Future studies should take this into consideration. Second, there was a lack of information about lens status and uveitis, known ocular risk factors for IOP elevation [36]. Third, the causal relationship between fatty liver and EIOP could not be verified due to the cross-sectional study design. Finally, the results of the current study may not be applicable to other races because we only analyzed single-center data on South Korean adults. Despite these limitations, we verified the relationship between fatty liver and EIOP using large population-based data. Furthermore, we identified that fatty liver contributes directly to IOP rather than indirectly to IOP via insulin resistance.

## 5. Conclusions

Fatty liver was an independent risk factor for EIOP. With increasing severity of fatty liver, the risk of EIOP became higher. The relationship between fatty liver and IOP was primarily linked directly rather than indirectly through insulin resistance. This suggests that it is necessary to assess the risk of EIOP in people with fatty liver, especially as the severity of fatty liver worsens or if ALD is a cause of fatty liver. Randomized controlled studies should be performed to identify the causal relationship between fatty liver and glaucoma development. Experimental studies are warranted to verify the mechanisms by which fatty liver affects IOP.

## Figures and Tables

**Table 1:** Clinical characteristics of the study population.

Variables	Non-Fatty Liver Group	Fatty Liver Group	Total Participants	p *
	(*n* = 10,454)	(*n* = 5786)	(*n* = 16,240)	
Male sex, *n* (%)	4637 (44.4%)	4229 (73.1%)	8866 (54.6%)	<0.001
Age, years	46.1 ± 12.5	46.7 ± 12.3	46.3 ± 12.4	0.002
BMI, kg/m^2^	22.5 ± 2.8	26.5 ± 3.3	24.0 ± 3.5	<0.001
WC, cm	80.5 ± 8.4	91.7 ± 9.3	84.5 ± 10.2	<0.001
MBP	86.6 ± 10.5	93.1 ± 10.7	88.9 ± 11.0	<0.001
Smoking status, *n* (%)				<0.001
Never-smoker	8170 (78.4%)	3663 (63.5%)	11833 (73.1%)	
Former smoker	806 (7.7%)	766 (13.3%)	1572 (9.7%)	
Current smoker	1449 (13.9%)	1342 (23.3%)	2791 (17.2%)	
Current drinker, *n* (%)	5526 (53.0%)	3280 (56.8%)	8806 (54.4%)	<0.001
Regular exerciser, *n* (%)	1938 (18.6%)	1047 (18.1%)	2985 (18.4%)	0.495
Hematocrit, %	42.3 ± 4.0	44.4 ± 3.9	43.1 ± 4.1	<0.001
FPG, mg/dL	87.1 ± 15.7	99.2 ± 26.5	91.4 ± 21.0	<0.001
HOMA-IR	0.8 ± 1.4	1.7 ± 1.8	1.1 ± 1.6	<0.001
HbA1c, %	5.5 ± 0.6	5.9 ± 1.0	5.6 ± 0.8	<0.001
Total cholesterol, mg/dL	190.2 ± 34.3	199.3 ± 39.3	193.5 ± 36.4	<0.001
AST, U/L	24.0 ± 13.7	30.5 ± 17.2	26.3 ± 15.4	<0.001
ALT, U/L	21.5 ± 16.8	38.0 ± 28.0	27.4 ± 22.9	<0.001
hsCRP, mg/dL	0.1 ± 0.3	0.2 ± 0.3	0.1 ± 0.3	<0.001
HBV, *n* (%)	327 (3.1%)	121 (2.1%)	448 (2.8%)	<0.001
HCV, *n* (%)	25 (0.2%)	15 (0.3%)	40 (0.2%)	0.934
DM, *n* (%)	451 (4.3%)	935 (16.2%)	1386 (8.5%)	<0.001
HTN, *n* (%)	1491 (14.3%)	1947 (33.7%)	3438 (21.2%)	<0.001
Dyslipidemia, *n* (%)	2498 (23.9%)	3225 (55.7%)	5723 (35.2%)	<0.001
IOP, mmHg	15.4 ± 2.9	16.3 ± 2.9	15.7 ± 2.9	<0.001
EIOP, *n* (%)	206 (2.0%)	249 (4.3%)	455 (2.8%)	<0.001

* *p* value for the comparison of the clinical characteristics between participants with fatty liver and those without fatty liver. Significance was set at *p* < 0.05. Abbreviations: BMI, body mass index; WC, waist circumference; MBP, mean blood pressure, FPG, fasting plasma glucose, HOMA-IR, homeostatic assessment model for insulin resistance; HbA1c, glycosylated hemoglobin; HDL, high-density lipoprotein; LDL, low-density lipoprotein; AST, aspartate aminotransferase; ALT, alanine aminotransferase; hsCRP, high-sensitivity C-reactive protein; HBV, hepatitis B virus; HCV, hepatitis C virus; DM; diabetes mellitus; HTN, hypertension; IOP, intraocular pressure; EIOP, elevated intraocular pressure.

**Table 2:** Logistic regression analysis to estimate the risk of elevated intraocular pressure according to fatty liver status.

EIOP	Unadjusted		Model 1		Model 2		Model 3	
	OR (95% CI)	*p* *	OR (95% CI)	*p* *	OR (95% CI)	*p* *	OR (95% CI)	*p* *
No fatty liver	1 (reference)		1 (reference)		1 (reference)		1 (reference)	
Fatty liver	2.24 (1.86–2.70)	<0.001	1.45 (1.16–1.80)	0.001	1.47 (1.18–1.84)	0.001	1.36 (1.08–1.71)	0.008

Model 1: adjusted for age, sex, and BMI. Model 2: adjusted for age, sex, BMI, smoking status, drinking status, and regular exercise. Model 3: adjusted for age, sex, BMI, smoking status, drinking status, regular exercise, hematocrit, history of DM, HTN, and dyslipidemia. * *p* value derived from logistic regression analysis. Significance was set at *p* < 0.05. Abbreviations: EIOP, elevated intraocular pressure; OR, odds ratio; CI, confidence interval; BMI, body mass index; DM; diabetes mellitus; HTN, hypertension.

**Table 3:** Logistic regression analysis for the risk of elevated intraocular pressure according to the etiology of fatty liver.

EIOP	Unadjusted		Model 1		Model 2		Model 3	
	OR (95% CI)	*p* *	OR (95% CI)	*p*-value	OR (95% CI)	*p* *	OR (95% CI)	*p* *
No fatty liver	1 (reference)		1 (reference)		1 (reference)		1 (reference)	
Fatty liver with viral hepatitis	1.90 (0.77–4.69)	0.164	1.10 (0.44–2.75)	0.846	1.07 (0.43–2.71)	0.879	1.01 (0.40–2.54)	0.991
ALD	2.55 (1.86–3.50)	<0.001	1.63 (1.17–2.27)	0.004	2.02 (1.43–2.85)	<0.001	1.80 (1.27–2.56)	0.001
NAFLD	2.18 (1.78–2.66)	<0.001	1.40 (1.10–1.77)	0.005	1.35 (1.07–1.72)	0.013	1.26 (0.99–1.61)	0.064

Model 1: adjusted for age, sex, and BMI. Model 2: adjusted for age, sex, BMI, smoking status, drinking status, and regular exercise. Model 3: adjusted for age, sex, BMI, smoking status, drinking status, regular exercise, hematocrit, DM, HTN, and dyslipidemia. * *p* value derived from logistic regression analysis. Significance was set at *p* < 0.05. Abbreviations: EIOP, elevated intraocular pressure; CI, confidence interval; ALD, alcoholic liver disease; NAFLD, nonalcoholic fatty liver disease; BMI, body mass index; DM, diabetes mellitus, HTN, hypertension.

## Data Availability

Not applicable.

## Supplementary Materials

The following supporting information can be downloaded at: https://www.mdpi.com/article/10.3390/nu14214455/s1. Figure S1. Mediation analysis on the effect of insulin resistance on intraocular pressure. Figure S2. Forest plot showing the OR (95% CI) for EIOP of ALD versus NAFLD and the OR (95% CI) for EIOP of drinkers versus nondrinkers.
