# Supplementary material for: Fatty Liver Is an Independent Risk Factor for Elevated Intraocular Pressure
Source: Nutrients. 2022 Oct 23;14(21):4455. doi: 10.3390/nu14214455 (PMC9657431; doi:10.3390/nu14214455)
Supplement: Supplementary file 1 [file nutrients-14-04455-s001.zip › nutrients-1977106-supplementary.pdf]

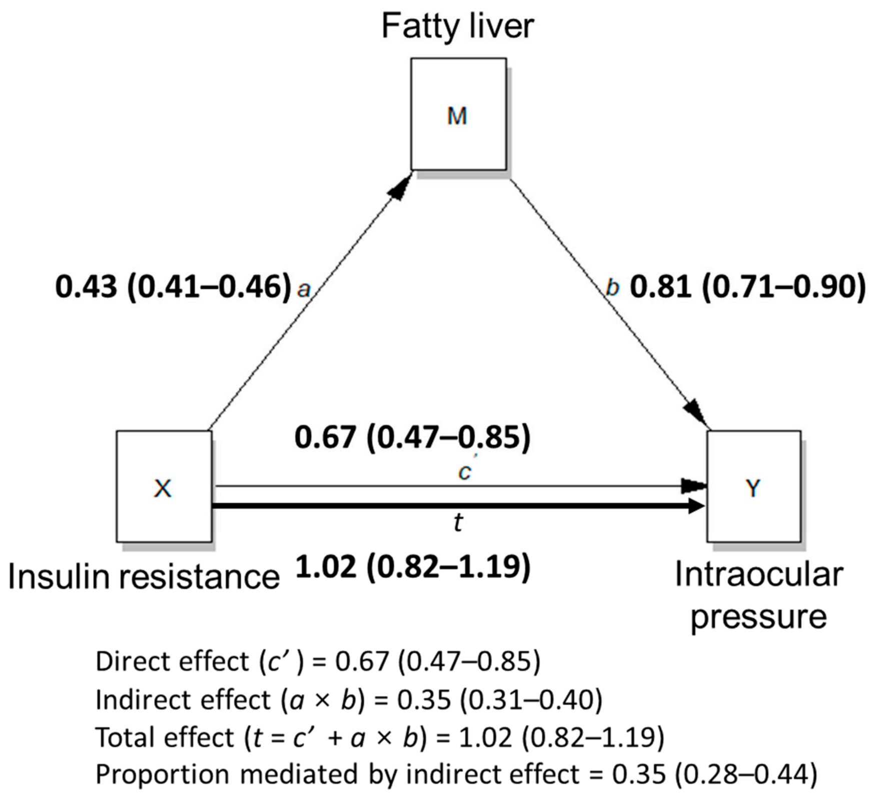

Supplementary Figure S1. Mediation analysis on the effect of insulin resistance on intraocular pressure. a, b, c and c' are path coefficients representing unstandardized regression weights and 95% confidence interval. *c′*, direct effect; *a* × *b*, indirect effect; *t*, total effect; X, dependent variable; M, mediator variable; Y, independent variable; IOP, intraocular pressure.

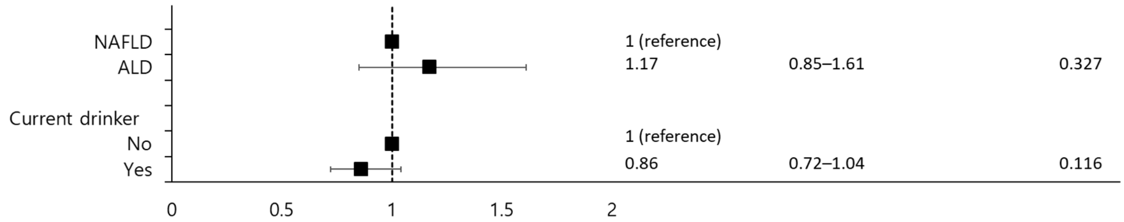

	Odds ratio for EIOP	95% Confidence interval	*p*
NAFLD	1 (reference)		
ALD	1.17	0.85–1.61	0.327
Current drinker			
No	1 (reference)		
Yes	0.86	0.72–1.04	0.116

Supplementary Figure S2. Forest plot showing the OR (95% CI) for EIOP of ALD versus NAFLD and the OR (95% CI) for EIOP of drinkers versus nondrinkers. Abbreviations: OR, odds ratio; CI, confidence interval; ALD, alcoholic liver disease; NAFLD, nonalcoholic fatty liver disease.
